# Type I Interferon Promotes Antitumor T Cell Response in CRPC by Regulating MDSC

**DOI:** 10.3390/cancers13215574

**Published:** 2021-11-08

**Authors:** Lilv Fan, Guiliang Xu, Jingjing Cao, Min Li, Huihui Zhang, Fanlin Li, Xinyue Qi, Xiaoqing Zhang, Zeyu Li, Ping Han, Xuanming Yang

**Affiliations:** 1 Sheng Yushou Center of Cell Biology and Immunology, School of Life Sciences and Biotechnology, Shanghai Jiao Tong University, Shanghai 200240, China; fanlilv@sjtu.edu.cn (L.F.); asdxgl@163.com (G.X.); jjcao@sjtu.edu.cn (J.C.); limin_1216@sjtu.edu.cn (M.L.); ninxia0@sjtu.edu.cn (H.Z.); lifanlin@sjtu.edu.cn (F.L.); xinyueqi@sjtu.edu.cn (X.Q.); zxq0318@sjtu.edu.cn (X.Z.); davidking2007@sina.com (Z.L.); esterhanping@sjtu.edu.cn (P.H.); 2 Joint International Research Laboratory of Metabolic & Developmental Sciences, Shanghai Jiao Tong University, Shanghai 200240, China

**Keywords:** IFNα, prostate cancer, G-MDSC, immunotherapy

## Abstract

**Simple Summary:**

Despite initial tumor regression following androgen blockade treatment, relapse of castration-resistant prostate cancer (CRPC) eventually occurs in most patients. Immunotherapy aims to activate the host immune system to fight against cancer and has achieved significant therapeutic effects in various solid tumors. The purpose of our research was to investigate the mechanisms underlying the immune response during CRPC development and to screen effective immunotherapies against CRPC. We found that interferon-α (IFNα) directly inhibited the progression of CRPC, reduced the accumulation of the immune suppressive granulocytic myeloid-derived suppressor cells (G-MDSCs) in the tumor microenvironment (TME), and impaired the inhibitory function of G-MDSCs on T cell activation. This research provides a potential strategy for the clinical treatment of CRPC.

**Abstract:**

Background: Metastatic castration-resistant prostate cancer (CRPC) is the leading cause of death among prostate cancer patients. Here, our aim was to ascertain the immune regulatory mechanisms involved in CRPC development and identify potential immunotherapies against CRPC. Methods: A CRPC model was established using Myc-CaP cells in immune-competent FVB mice following castration. The immune cell profile of the tumor microenvironment (TME) was analyzed during CRPC development. Different immunotherapies were screened in the CRPC tumor model, and their efficacies and underlying mechanisms were investigated in vitro and in vivo. Results: During CRPC development, the proportion of granulocytic myeloid-derived suppressor cells (G-MDSCs) in the TME increased. Among the immunotherapies tested, IFNα was more effective than anti-PD-L1, anti-CTLA-4, anti-4-1BB, IL-2, and IL-9 in reducing Myc-CaP CRPC tumor growth. IFNα reduced the number of G-MDSCs both in vitro during differentiation and in vivo in CRPC mice. Furthermore, IFNα reduced the suppressive function of G-MDSCs on T cell proliferation and activation. Conclusion: G-MDSCs are crucial to effective immunotherapy against CRPC. Treatment with IFNα presents a promising therapeutic strategy against CRPC. Besides the direct inhibition of tumor growth and the promotion of T cell priming, IFNα reduces the number and the suppressive function of G-MDSCs and restores T cell activation.

## 1. Introduction

Prostate cancer is the second most commonly diagnosed cancer among men worldwide [1]. The incidence of prostate cancer is related to factors such as age, genetics, and ethnicity [2,3]. As the tumor grows, it spreads to tissues such as bone and lymph nodes. Surgery and radiotherapy, the most common treatment strategies for prostate cancer, have side effects, such as incontinence and sexual dysfunction [4]. The growth and development of prostate cancer were considered to be associated with androgens [5]. Consequently, androgen deprivation has been widely used clinically as an effective treatment strategy for prostate cancer. In addition to surgical methods, certain chemical castration drugs, such as abiraterone acetate, an inhibitor of androgen synthesis, and enzalutamide, an inhibitor of androgen receptors, have also been used [6,7]. However, about 20–40% of metastatic CRPC patients show no response to the drugs used for the treatment of castration-resistant tumors, eventually resulting in low drug efficacy among all men [8].

Compared to conventional therapies (chemotherapy and surgery), immune therapies, such as immune checkpoint inhibitors, cytokines, cellular immunotherapy, etc., have been proven to possess improved clinical therapeutic effects against many tumors. Interferons (IFNs) are a family of cytokines that directly act on tumor cells or indirectly activate the immune response to resist invasion by cancer cells [9]. IFNα belongs to the type I IFN family. Its receptor is composed of two subunits: IFNAR1 and IFNAR2 [10]. Most cells in the body secrete and respond to IFNα. It plays an important role in the immune response against viruses and tumors. IFNα, through the JAK-STAT signaling pathway, induces the translocation of STAT1/2 heterodimers or STAT1 homodimers into the nucleus and drives the expression of IFN-related genes [11,12]. It regulates numerous genes associated with tumor proliferation, survival, and migration [13]. In vitro, IFNα upregulates p21 in prostate cancer cells and slows down the cell cycle [14]. In addition to its direct effects on tumors, IFNα also regulates the antitumor immune response of almost all types of immune cells. It improves the generation and survival of tumor-specific T cells [15], enhances the cytotoxicity of natural killer (NK) cells to tumors [16], increases survival and promotes the antibody response of B cells [17], induces the maturation of dendritic cells (DCs) and enhances their ability to cross-present tumor-associated antigens to CD8^+^ T cells [18,19], negatively regulates the proliferation of regulatory T cells (Tregs) [20], and induces the antigen-presenting cell (APC)-mediated expression of IL-15, which is critical for the proliferation and maintenance of lymphocytes [21].

There are multiple immune suppressive cell subtypes in the solid tumor microenvironment (TME). Treg, myeloid-derived suppressor cells (MDSCs), and tumor-associated macrophages (TAMs) in the TME inhibit the functions of immune effector cells such as T cells and NK cells [22,23]. MDSCs are derived from the bone marrow. In cancer patients, the tumor cells secrete cytokines, such as GM-CSF, G-CSF, and IL-6 [24], and result in the hematopoietic stem cells in the bone marrow abnormally developing into MDSCs. The tumor cells also secrete CCLs and CXCLs and other chemokines, then recruit MDSCs to the TME to exert immunosuppressive functions [25]. MDSCs reduce the level of L-arginine, which is required for the proliferation of T cells in the TME, through Arg1, and produce NO, ROS, and peroxynitrite (PNT), which inhibit T cells from entering the TME [26]. They also produce immunosuppressive cytokines, such as IL-10 and TGF-β, which induce the generation of Tregs [27,28]. MDSCs are divided into two subgroups: monocytic MDSCs (M-MDSCs) and granulocytic MDSCs (G-MDSCs). Although both M-MDSCs and G-MDSCs possess immunosuppressive functions, the ratio of G-MDSCs/M-MDSCs of different tumors is quite different [29].

In this study, we detected the changes in the TME during CRPC development. We found that the proportion of G-MDSCs increased significantly. We also tested the effectiveness of several immune checkpoint inhibitors and cytokines for the treatment of CRPC and found that IFNα treatment inhibited tumor growth and reduced the accumulation of G-MDSCs in the TME. This antitumor effect of IFNα depended on the CD8^+^ T cells. Furthermore, both in vivo and in vitro, IFNα significantly reduced the number of G-MDSCs in the bone marrow. In vitro, it significantly decreased the immunosuppressive function of G-MDSCs on T cells by upregulating the expression of immune-promoting molecules. This work provides a potential strategy for the treatment of CRPC.

## 2. Materials and Methods

### 2.1. Mice

FVB mice were purchased from Shanghai Lingchang Biotechnology Co., Ltd. *Ifnar1*^−/−^ mice were kindly provided by Dr. Anita Chong from the University of Chicago. *Ifnar1*^−/−^ FVB mice were generated by crossing the *Ifnar1*^−/−^ C57BL/6 mice and the FVB mice for 6 generations.

### 2.2. Cell Lines and Reagents

Myc-CaP is a prostate cancer cell line derived from the spontaneous prostate tumor in mice [30]. Myc-CaP cells were cultured in Dulbecco’s Modified Eagle Medium (DMEM) supplemented with 10% heat-inactivated fetal bovine serum (FBS) (Gibco, Paisley, UK), 100 units/mL penicillin, 100 μg/mL streptomycin, 2 mM L-glutamine, and 55 μM β-mercaptoethanol. Cells were cultured in an incubator at 37 °C with 5% CO_2_.

### 2.3. Myc-CaP Treatment In Vitro

A total of 3 × 10^4^ Myc-CaP cells were seeded in 24 well plates with DMEM complete culture medium supplemented with PBS or IFNα4 (50 or 200 ng/mL). After 48 h, the cells were digested with trypsin, and the number of cells was counted using a hemocytometer.

### 2.4. MDSCs Differentiation from Bone Morrow Cells

Femurs and tibias were obtained from male FVB mice, and the bone marrow cavities were flushed with PBS using an insulin syringe. ACK (Ammonium-Chloride-Potassium) lysis buffer (BD Biosciences, San Jose, CA, USA) was used to lyse the red blood cells in all samples. Bone marrow cells were cultured in Petri dishes containing RPMI-1640 complete culture medium supplemented with 20 ng/mL GM-CSF (Sinobiological Catalog No: 51048-MNAH, Beijing, China) and induced for 4 days by treating with PBS or IFNα4 (20 ng/mL or 100 ng/mL), to generate bone marrow-derived MDSCs (BM-MDSCs). On day 4, the proportion of G-MDSCs was detected by flow cytometry, and the cell number was obtained using a hemocytometer.

### 2.5. Isolation of G-MDSCs

Bone marrow cells were induced for 4 days. The single-cell suspension was incubated with 2.4G2 (antibodies recognizing CD16 and CD32) for 10 min to block non-specific Fc-mediated binding. The BM-MDSCs were magnetically labeled with anti-Ly-6G-Biotin (1A8) (Biolegend, San Diego, CA, USA) and Streptavidin Nanobeads (Biolegend). Subsequently, they were separated on a magnetic rack to obtain G-MDSCs.

### 2.6. T Cell Inhibition by G-MDSCs

Mouse spleens were ground and passed through a 70 μm cell strainer. Red blood cells were lysed using ACK lysis buffer and isolated using the MojoSort™ Mouse CD3 T Cell Isolation Kit (BioLegend), according to the manufacturer’s instructions. The purified T cell suspension was incubated in the dark with 5 µM carboxyfluorescein succinimidyl ester (CSFE) (Selleck, Boston, MA, USA) for 7 min and washed twice with RPMI-1640 complete culture medium to remove the unbound CFSE. Subsequently, they were co-cultured with Ly6G^+^ BM-MDSCs in the ratio of 1:1 and 3:1 in 96-well plates with RPMI-1640 complete culture medium (10% heat-inactivated FBS, 100 units/mL penicillin, 100 μg/mL streptomycin, 2 mM L-glutamine, and 55 μM β-mercaptoethanol). Plate-bound anti-CD3 (0.5 µg/mL) and anti-CD28 (1 µg/mL) antibodies were used to stimulate the T cells in the culture. Forty-eight hours after activation, the cells and supernatants were collected for flow cytometry and cytometric bead array (CBA) analyses.

### 2.7. Isolation of Single Cells from Tumors

Mouse tumor tissues were sliced to pieces using surgical scissors and digested in tumor dissociation buffer (RPMI-1640 with 50 μg/mL Liberase TL (Roche) and 200 μg/mL DNase Ι (Sigma, St Louis, MO, USA)). The tumor tissues were ground and passed through a 70 μm cell strainer. The single cells obtained were re-suspended in staining buffer (1 × PBS with 1% FBS).

### 2.8. Flow Cytometry Analysis

Single-cell suspensions of cells were incubated with 2.4G2 for 10 min. Blocked samples were subsequently stained with fluorescently labeled monoclonal antibodies and a fluorescent intercalator. The anti-mouse CD45-APC/Cyanine7 (30-F11 Catalog No: 103116), anti-mouse Ly-6C-FITC (HK1.4 Catalog No: 128006), anti-mouse CD11c-PE (N418 Catalog No: 117308), anti-mouse CD4-FITC (GK1.5 Catalog No: 100406), anti-mouse CD8a-AF700 (53-6.7 Catalog No: 100730), anti-mouse CD19-PE (6D5 Catalog No: 115508), anti-mouse CD335-APC (29A1.4 Catalog No: 137608), anti-mouse CD4-APC/Cyanine7 (GK1.5 Catalog No: 100414), anti-mouse CD8a-Pacific Blue (53-6.7 Catalog No: 100725), and anti-mouse CD45-Pacific Blue (30-F11 Catalog No: 103126) antibodies were purchased from BioLegend. 7-AAD (Catalog No: 559925) was purchased from BioLegend. The anti-mouse CD11b-AF700 (M1/70 Catalog No: 56-0112-82), anti-mouse Ly-6G-APC (1A8 Catalog No: 17-9668-82), and anti-mouse CD11b-APC (M1/70 Catalog No: 17-0112-81) antibodies were purchased from eBioscience. The anti-Mouse Ly-6G-FITC (1A8 Catalog No: 551460) antibody was purchased from BD Pharmingen. The samples were evaluated on a CytoFLEX S Flow Cytometer (Beckman Coulter, Suzhou, Jiangsu, China), and the results were analyzed using the FlowJo software (TreeStar, version 10.0.7, Ashland, OR, USA).

### 2.9. Cytokine Production Analysis

Production of the cytokine, IFN-γ, in co-culture supernatants from the T cells and Ly6G^+^ BM-MDSCs was tested using the Cytometric Bead Array Kit (BD biosciences), according to the manufacturer’s instructions.

### 2.10. RT-qPCR

Total RNA was extracted using the E.Z.N.A.^®^ Total RNA Kit I (Omega Bio-Tek, Norcross, GA, USA) and reverse transcribed using the GoScript Reverse Transcription system (Promega, Madison, WI, USA). Specific gene was amplified using 2 × ChamQ Universal SYBR qPCR Master Mix (Vazyme, Nanjing, Jiangsu, China) and quantified by real-time PCR, according to manufacturer’s instructions. The qPCR primers are enlisted in Supplementary Materials Table S1.

### 2.11. Mouse CRPC Model and Immunotherapy

On day -14, four- to six-week-old male FVB mice were transplanted with 3 × 10^6^ Myc-CaP cells by subcutaneous injection. On day 0, following the implantation of tumor cells, the mice were castrated by surgery. The mice were treated with 20 μg human IgG, mouse IFNα4 (produced in house), anti-mouse PD-L1 antibody (Bioxcell, clone 10F.9G2, Lebanon, NH, USA), anti-mouse CTLA-4 antibody (produced in house), anti-mouse 4-1BB antibody (Bioxcell, clone 3H3), mouse IL-2 (produced in house), or mouse IL-9 (produced in house) on days 14, 17, and 21, by intratumoral injection. For cell depletion experiments, 200 μg YTS.169.4.2 (anti-mouse CD8 antibody, produced in house), GK1.5 (anti-mouse CD4 antibody, produced in house), or PK136 (anti-mouse NK1.1 antibody, produced in house) on days 14 and 17, by intraperitoneal injection.

### 2.12. Statistical Analysis

The data were analyzed using the GraphPad Prism 8 software (GraphPad Software Inc, La Jolla, CA, USA). The significance of assays was determined using the unpaired Student’s two-tailed *t*-test. Where indicated, * *p* < 0.05, ** *p* < 0.01, *** *p* < 0.001 were considered as statistically significant results.

## 3. Results

### 3.1. G-MDSCs Are Increased in Prostate TME Following Castration

**Figure 1:**
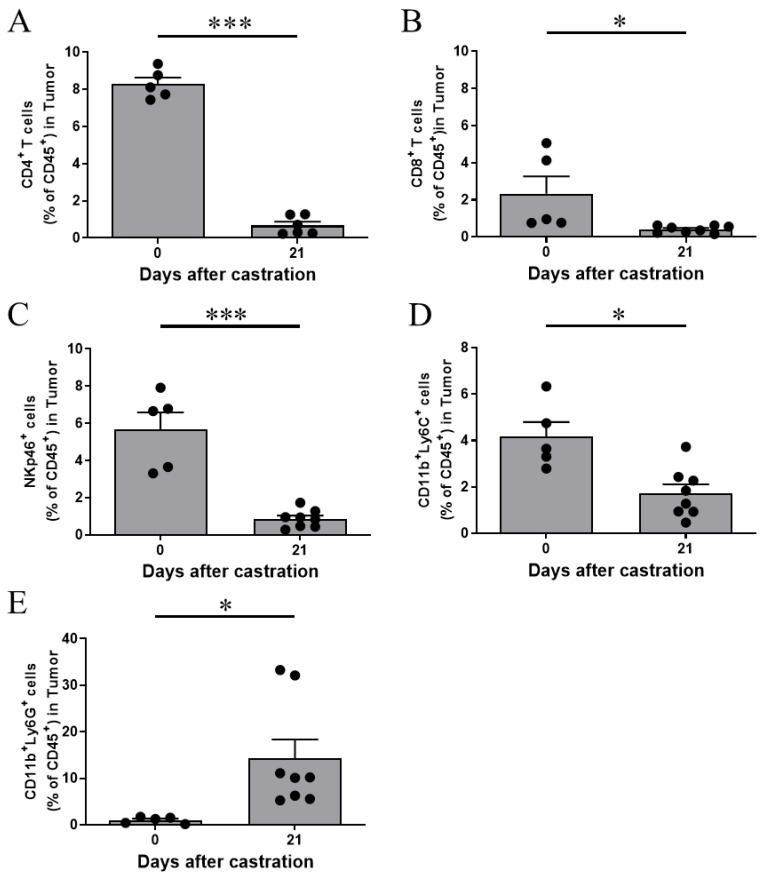
Analysis of the different types of immune cells in the tumor microenvironment (TME) after castration. (**A**–**E**) Male FVB mice were subcutaneously injected with 3 × 10^6^ Myc-CaP cells over the right flank on day -14 and castrated by surgery on day 0. Specific immune cells in the tumor were analyzed by flow cytometry on days 0 and 21 after castration. Representative bar graphs of the flow cytometry analyses of CD4^+^ T cells (**A**), CD8^+^ T cells (**B**), NK cells (**C**), M-MDSCs (**D**), and G-MDSCs (**E**) in CD45^+^ immune cells in the tumor. Statistical significance was determined by unpaired *t*-test and is represented by * *p* < 0.05, *** *p* < 0.001. Pooled results from two replicates have been shown in (**A**–**E**) (mean ± SEM), *n* = 5–8 per group.

Although androgen deprivation therapies are initially effective against prostate cancer, resistance and relapse occur eventually. During relapse, the prostate tumor cells alter their growth pattern to an androgen-independent manner. The changes in the immune cells found in the TME during the transition of prostate cancer from an androgen-dependent (AD) to a castration-resistant (CR) form remain unclear. We established a Myc-CaP prostate tumor model in immune-competent FVB mice. Following castration, we found that the Myc-CaP prostate tumor underwent remission initially and relapsed later, which resembled the clinical development of castration-resistant prostate cancer [31]. Following the castration of the tumor-bearing mice, we analyzed the immune cell composition in the TME at different time points in both the remission and relapse periods. We found that the infiltration of CD8^+^ T cells, CD4^+^ T cells, and NK cells significantly decreased during the remission and relapse of prostate cancer (Figure 1A–C). These results suggest the inability or weak ability of these cells to induce antitumor immunity after castration. Interestingly, a subtype of MDSCs, G-MDSCs, were significantly enriched in the TME in the relapse periods after castration (Figure 1E), but M-MDSCs decreased in the TME (Figure 1D), suggesting that G-MDSCs may be one of the primary reasons for the suppression of antitumor immunity and promotion of CRPC development.

### 3.2. IFNα Was Effective in Controlling CRPC

**Figure 2:**
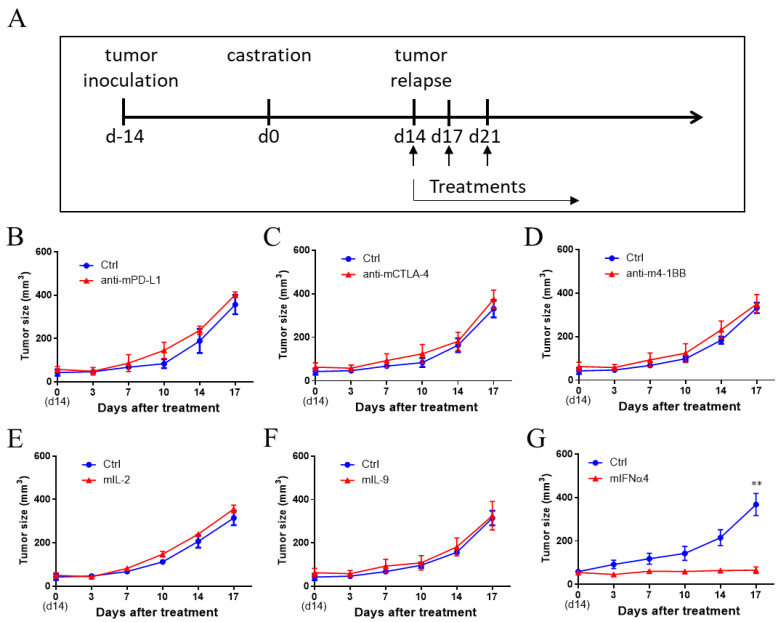
Therapeutic effect of different immunotherapies in CRPC mouse model. (**A**) Treatment schedule using different immunotherapies in Myc-CaP CRPC-bearing mice. Myc-CaP cells (3 × 10^6^ cells/mouse) were subcutaneously inoculated over the right flank of 4–6-week-old male FVB mice. Fourteen days after inoculation, the mice were castrated by surgery. Fourteen days after castration, the tumor relapsed. The mice were administered an intratumoral injection of immune checkpoint inhibitors or cytokines (20 μg/mouse) on days 14, 17, and 21. The tumor volumes were measured using a vernier caliper twice a week and calculated using the formula: (length × width × height)/2. (**B**–**G**) Fourteen days after castration, the mice were treated with control (hIgG), anti-mouse-PD-L1 (**B**), anti-mouse-CTLA-4 (**C**), anti-mouse-4-1BB (**D**), mouse IL-2 (**E**), mouse IL-9 (**F**), or mouse IFNα4 (**G**). Statistical significance was determined using the unpaired *t*-test and is represented by ** *p* < 0.01. Representative results from one of one or two replicates are shown (**B**–**G**) (mean ± SEM), *n* = 4–5 per group.

T cells are critical for conferring antitumor immunity. Immune checkpoint blockade antibodies targeting T cells have shown promising antitumor effects in both preclinical models as well as patients with cancer [32,33]. Since T cell infiltration was decreased in the TME after castration, we examined whether immune checkpoint antibodies could re-activate T cells to reduce the tumor burden during CRPC. For this, we combined anti-PD-L1 or anti-CTLA-4 treatment with castration. However, these two immune checkpoint antibodies showed no antitumor effects during CRPC (Figure 2A–C). Insufficient co-stimulation signal and T cell proliferation cytokines are possible mechanisms for weak T cell activation in the TME [34]. Subsequently, we combined castration with anti-4-1BB co-stimulation agonist antibody and cytokine IL-2 or IL-9. Anti-4-1BB antibodies have shown strong antitumor activity in various tumor models, including immune checkpoint blockade antibody-resistant tumor models [35,36,37,38]. The cytokine IL-2 is critical for T cell proliferation and has shown antitumor activity in both preclinical models and in patients with cancer [39]. IL-9 not only induces innate and adaptive immune responses but also directly promotes tumor apoptosis [40]. However, anti-4-1BB antibody, IL-2, and IL-9 failed to reduce tumor burden in the Myc-CaP CRPC tumor model (Figure 2D–F). Recent studies have shown that type I interferons are critical for the generation of antitumor T cell immunity [41]. Interestingly, IFNα4 treatment showed potent antitumor activity when combined with castration (Figure 2G). Since all five types (anti-PD-L1, anti-CTLA-4, anti-4-1BB, IL-9, and IL-2) of T cell-targeting treatments were not effective against CRPC, it suggests that IFNα treatment may generate antitumor immunity through non-T cells, such as antigen-presenting cells and immune suppressive cells, which are critical for generating antitumor immunity.

### 3.3. IFNα4 Reduced Immunosuppression in the TME

**Figure 3:**
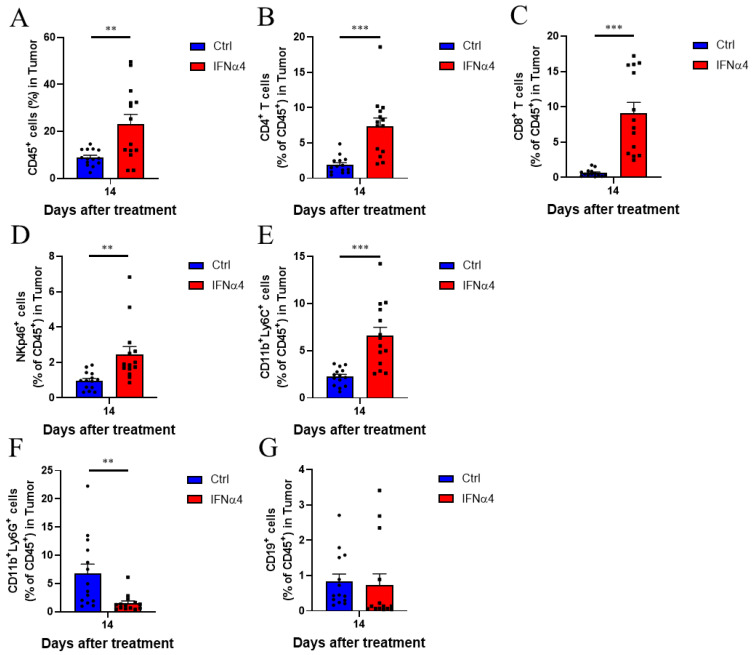
IFNα4 altered the immune cell components in the TME. (**A**–**G**) Male FVB mice were subcutaneously injected with Myc-CaP cells (3 × 10^6^ cells/mouse) on day -14 and castrated by surgery on day 0. The mice were administered an intratumoral injection of 20 μg of hIgG or IFNα4 on days 14, 17, and 21. The immune cells in the tumor tissue were analyzed by flow cytometry on day 14 post hIgG or IFNα4 treatment. Flow cytometry analysis of CD45^+^ immune cells (**A**), CD4^+^ T cells (**B**), CD8^+^ T cells (**C**), NK cells (**D**), M-MDSCs (**E**), G-MDSCs (**F**), and B cells (**G**) in the tumor are shown. Statistical significance was determined using unpaired *t*-test and is represented by ** *p* < 0.01, *** *p* < 0.001. Pooled results from four replicates have been shown in (**A**–**G**) (mean ± SEM), *n* = 14 per group.

Since our data indicated that the T cell targeting treatment through immune checkpoint blockade, co-stimulation enhancement, and T cell growth stimulation was not sufficient to activate efficient antitumor T cell immune response, we hypothesized that non-T immune cells may be critical for IFNα-mediated tumor suppression. To verify this, we analyzed the immune cell profile in the TME of castrated Myc-CaP bearing mice on day 14 post IFNα4 treatment. We found that the infiltration of CD45^+^ leukocytes in the tumor tissue was significantly increased on day 14 (8.9% vs. 23%) post IFNα4 treatment (Figure 3A). Among the CD45^+^ leukocytes, IFNα4 treatment had little effect on the infiltration of CD19^+^ B cells (Figure 3G). However, it significantly increased the infiltration of CD4^+^ T cells (1.9% vs. 7.4%), CD8^+^ T cells (0.6% vs. 9.1%), and NK cells (1% vs. 2.5%) on day 14 post treatment (Figure 3B–D). These findings are consistent with the reduced tumor burden following IFNα4 treatment. In addition, we also observed a significant decrease in the proportion of immune suppressive G-MDSCs on day 14 after IFNα4 treatment (6.8% vs. 1.5%) (Figure 3F), but not M-MDSCs in the TME (2.2% vs. 6.6%) (Figure 3E). These data suggest that IFNα4 acts on multiple immune cell types to simultaneously promote cells with tumor-killing effect and reduce tumor-promoting immune suppressive cells.

### 3.4. Cytotoxic T Cells Are Critical for IFNα-Mediated Therapeutic Effect on CRPC

**Figure 4:**
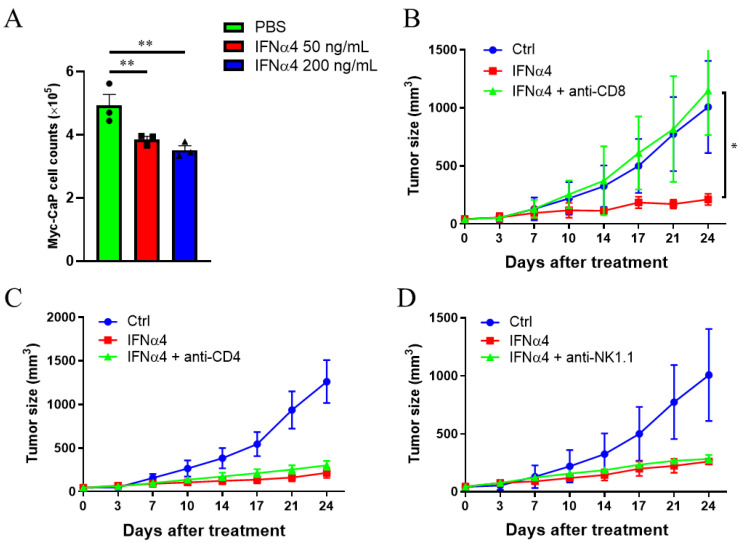
IFNα4 reduced tumor burden by directly inhibiting tumor cell growth and promoting the antitumor T cell response. (**A**) A total of 3 × 10^4^ Myc-CaP cells were seeded in 24 well plates and treated with PBS or IFNα4 (50 ng/mL or 200 ng/mL) for 48 h. The cell count was determined and compared. Statistical significance was determined using unpaired *t*-test and is represented by ** *p* < 0.01. Representative results from one of two replicates are shown (**A**) (mean ± SEM), with triplicate wells per group. (**B**–**D**) CRPC-bearing mice were treated with anti-CD8 (**B**), anti-CD4 (**C**), or anti-NK1.1 (**D**) Antibodies during IFNα4 treatment. Similar to Figure 2, mice were treated with 20 μg hIgG (Ctrl), 20 μg IFNα4, or 20 μg IFNα4 and 200 μg of the aforementioned antibodies. Statistical significance was determined using unpaired *t*-test and is represented by * *p* < 0.05. Representative results from one of one or two replicates are shown (**B**–**D**) (mean ± SD), *n* = 4–5 per group.

IFNAR is widely expressed on almost all cell types, including tumor and non-tumor cells, which are potential targets of IFNα treatment. Besides their direct inhibition of tumor growth, recent studies have highlighted the importance of IFNα in activating various immune cells, including T cells and NK cells [42]. In this study, first, we tested the direct effect of IFNα on Myc-CaP cells. Consistent with previous findings [14], IFNα4 significantly reduced Myc-CaP cell growth to about 70% in vitro (Figure 4A). Since CD4^+^ T cells, CD8^+^ T cells, and NK cells are crucial antitumor components and potential targets of IFNα, we tested if these cells were required for the IFNα-mediated tumor suppression of CRPC. We administered CD8^+^ T cell-, CD4^+^ T cell-, or NK cell-depleting Ab during the IFNα4 treatment of castrated Myc-CaP bearing FVB mice and measured tumor growth. CD8^+^ T cell depletion, and not CD4^+^ T cell or NK cell depletion, abolished the therapeutic effect of IFNα4 (Figure 4B–D). These data suggest that the antitumor activity of IFNα4 is mediated primarily through the activation of CD8^+^ T cell response.

### 3.5. IFNα4 Inhibited the Differentiation or Proliferation of G-MDSCs

**Figure 5:**
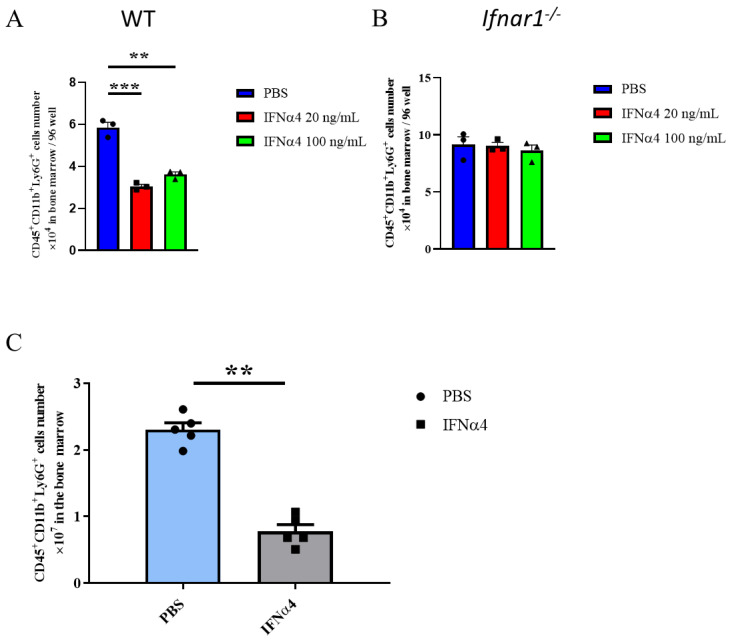
IFNα4 affected the growth of bone marrow-derived G-MDSCs. (**A**,**B**) A total of 1 × 10^5^ bone marrow cells from WT mice (**A**) or *Ifnar1^−/−^* mice (**B**) were differentiated with 20 ng/mL GM-CSF, combined with PBS or IFNα4 (20 ng/mL or 100 ng/mL) for 4 days, in 96-well plates. The G-MDSCs (CD45^+^CD11b^+^Ly6G^+^) cell number was compared. Statistical significance was determined using unpaired *t*-test and is represented by ** *p* < 0.01, *** *p* < 0.001. Representative results from one of two replicates are shown (**A**,**B**) (mean ± SEM), with triplicate wells per group. (**C**) Similar to Figure 2, Myc-CaP CRPC-bearing mice were treated with PBS or IFNα4 on days 14 and 17 after castration. On day 20, bone marrow cells were harvested, and the G-MDSCs (CD45^+^CD11b^+^Ly6G^+^) cell number was compared. Each point represents one mouse. Statistical significance was determined using unpaired *t*-test and is represented by ** *p* < 0.01. Representative results from one of two replicates are shown (**C**) (mean ± SEM), *n* = 5 per group.

Our results showed that IFNα4 reduced the accumulation of G-MDSCs and enhanced the antitumor T cell response. However, the mechanisms underlying the IFNα-mediated reduction in the accumulation of G-MDSCs, and the contribution of this reduction to enhanced T cell response remained unclear. To verify this, first, we tested if IFNα4 affected the proliferation of G-MDSCs. We performed in vitro differentiation of G-MDSCs from bone marrow precursor cells and found that the yield of G-MDSCs was significantly reduced (5.85 × 10^4^ vs. 3.0 × 10^4^) when IFNα4 was present, suggesting that IFNα4 directly affects G-MDSCs differentiation and proliferation (Figure 5A). To further confirm this, we obtained G-MDSCs from *Ifnar1^−/−^* bone marrow cells, which lack the ability to transduce the interferon signaling pathway. We found that IFNα4 had no effect on *Ifnar1^−/−^* G-MDSCs differentiation and proliferation (Figure 5B). To further confirm that IFNα4-mediated downstream signal activation was critical for G-MDSCs differentiation and proliferation, we investigated whether IFNα4 possessed the same function in vivo. Three days post the second treatment of CRPC-bearing mice with IFNα4. We observed a decrease in the number of G-MDSCs in the bone marrow (Figure 5C). Collectively, these results demonstrate that IFNα4 negatively regulates G-MDSCs differentiation and proliferation both in vitro and in vivo.

### 3.6. IFNα4 Inhibited the Immune Suppressive Function of G-MDSCs

**Figure 6:**
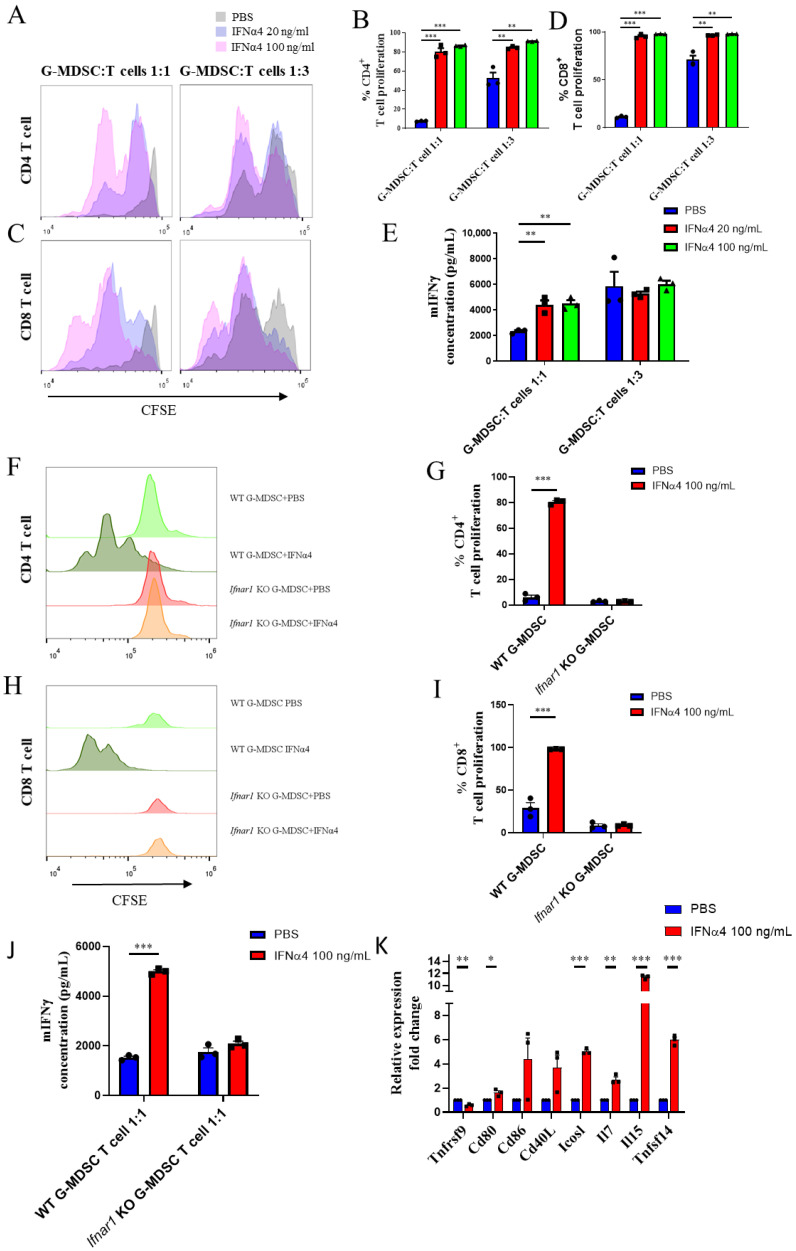
IFNα4 inhibited the suppressive function of G-MDSCs on T cell proliferation and activation. (**A**–**E**) Bone marrow cells from WT mice were differentiated with 20 ng/mL GM-CSF combined with PBS or IFNα4 (20 ng/mL or 100 ng/mL) for 4 days, and the G-MDSCs obtained were purified using anti-Ly6G magnetic beads. A total of 1 × 10^5^ CFSE-labeled, purified CD3^+^ T cells were co-cultured with purified G-MDSCs in a 1:1 and 3:1 ratio, respectively, in the presence of coated anti-CD3 and anti-CD28 antibodies. Forty-eight hours later, the proliferation of CD4^+^ T cells and CD8^+^ T cells was measured by CFSE dilution using flow cytometry (**A**–**D**). The concentration of the T cell effector cytokine, IFN-γ, in the supernatant was measured using the CBA kit (**E**). (**A**,**C**) represent the CD4^+^ or CD8^+^ T cell CFSE dilution histogram. (**B**,**D**) represent the percentage of divided cells in total CD4^+^ or CD8^+^ T cells. Statistical significance was determined using unpaired *t*-test and is represented by ** *p* < 0.01, *** *p* < 0.001. Representative results from one of two replicates are shown (**A**–**E**) (mean ± SEM), with triplicate wells per group. (**F**–**J**) WT or *Ifnar1^−/−^* mice-derived bone marrow cells were differentiated with 20 ng/mL GM-CSF combined with PBS or 100 ng/mL IFNα4 for 4 days, and the G-MDSCs obtained were purified using anti-Ly6G magnetic beads. Similar to panel (**A**–**E**), WT or *Ifnar1^−/−^* G-MDSCs were co-cultured with CFSE-labeled, purified T cells in a 1:1 ratio. The CFSE dilution of CD4^+^ T cells and CD8^+^ T cells (**F**–**I**) and the release of the effector cytokine, IFN-γ (**J**), were analyzed. Statistical significance is represented by *** *p* < 0.001. Representative results from one of two replicates are shown (**F**–**J**) (mean ± SEM), with triplicate wells per group. (**K**) WT mice-derived bone marrow cells were differentiated with 20 ng/mL GM-CSF combined with PBS or 100 ng/mL IFNα4 for 4 days. The G-MDSCs obtained were purified using anti-Ly6G magnetic beads and subjected to RNA isolation. The expression profile of T cell activation-related genes was analyzed by RT-qPCR. GAPDH was used as a housekeeping gene to normalize gene expression. Statistical significance was determined using unpaired *t*-test and is represented by * *p* < 0.05, ** *p* < 0.01, *** *p* < 0.001. Representative results from one of two replicates are shown (**K**) (mean ± SEM).

To test whether IFNα4 affected the immunosuppressive function of G-MDSCs, we performed magnetic bead sorting on in vitro differentiated G-MDSCs. An equal number of purified G-MDSCs were co-cultured with purified CFSE-labeled CD3^+^ T cells to evaluate the suppressive function of G-MDSCs on T cell activation and proliferation. We found that the inhibitory effect of G-MDSCs on both CD4^+^ T cell and CD8^+^ T cell proliferation was significantly reduced post exposure to IFNα4 (Figure 6A–D). Furthermore, to test whether IFNα4-treated G-MDSCs influenced the effector function of T cells, we analyzed the secretion of the T cell effector molecule, IFN-γ, in the co-culture supernatant; this revealed the activation status of T cells. Consistent with the reduced inhibitory effect of G-MDSCs on T cell proliferation, IFNα4-treated G-MDSCs showed a weaker inhibitory effect on IFN-γ secretion from T cells following their activation (Figure 6E). In addition, we also tested the immune suppressive function of IFNα4-treated *Ifnar1^−/−^* G-MDSCs. We found that the IFNα4-mediated reduction in the immune suppressive function of G-MDSCs was abolished in *Ifnar1^−/−^* G-MDSCs, indicating that the activation of downstream signaling pathways is crucial for the function of IFNα (Figure 6F–J). To elucidate the detailed regulatory mechanism of the effect of IFNα4 on G-MDSCs, we analyzed the mRNA expression profile of molecules associated with T cell activation by G-MDSCs cells. We observed a significant increase in the expression levels of co-stimulatory molecules such as ICOSL, TNFSF14, and CD40L, and T cell growth factors such as IL-7 and IL-15 (Figure 6K). These data suggest that IFNα4 not only inhibits the proliferation of G-MDSCs but also affects its immune suppressive function.

## 4. Discussion

Prostate cancer usually depicts slow growth. Detection and treatment before symptoms appear often results in limited improvement in the health and survival of patients. Clinically, surgical resection, hormonal therapy, and radiation therapy are used to treat prostate cancer. Androgen deprivation drugs, such as abiraterone or enzalutamide, cause anemia, lower bone density, and CRPC. Once patients develop CRPC, they become resistant to most therapeutic drugs, thereby limiting the treatment option to a few drugs [43]. In recent decades, immunotherapy has been increasingly used for the clinical treatment of prostate cancers. It prolongs the survival of patients and has fewer side effects. The tumor vaccine, sipuleucel-T, which targets prostatic acid phosphatase (PAP), has received U.S. FDA approval to be used in the treatment of metastatic CRPC (asymptomatic/minimally symptomatic) [44]. Moreover, several PSMA-directed CAR-T cells have undergone clinical trials for the treatment of metastatic CRPC, and the drugs have been identified as safe and feasible when used at the appropriate dosage [45,46]. Previous studies have shown that immune checkpoint inhibitors such as anti-PD-1 and anti-CTLA-4 slightly slow down the growth of CRPC. However, when they are combined with MDSCs depleting anti-Gr1 antibody or BEZ235 (dual PI3K and mTOR inhibitor), they significantly inhibit the growth of CRPC [47]. In addition, preclinical studies have shown that anti-IL-23 antibody inhibits the growth of CRPC and increases the efficacy of enzalutamide in the treatment of CRPC [48].

In this study, we focused on understanding the changes in the immune cell profile during CRPC development and designing potential immunotherapies for the treatment of CRPC. Various mechanisms trigger the development of CRPC: the increased sensitivity of the androgen receptor (AR) pathway or AR mutations lead to androgen-independent AR activation [49]. Besides these intrinsic changes in the tumor, our results showed that the changes in the immune cell profile of the TME might also contribute to CRPC development. We found a reduction in the infiltration of CD8^+^ T cells and NK cells and an increase in the proportion of immunosuppressive cells such as G-MDSCs, which resulted in increased immunosuppressive ability. To treat CRPC, we evaluated several immunotherapies, including immune checkpoint inhibitors such as anti-PD-L1 Ab and anti-CTLA-4 Ab, agonistic antibodies of co-stimulatory molecules, such as anti-4-1BB Ab, and cytokines, such as IL-2, IL-9, and IFNα4. These treatments have shown potent antitumor activity in some tumor models [38,50,51,52,53,54]. However, we found that only IFNα4 reduced the tumor burden in the Myc-CaP CRPC tumor model. IFNα directly acts on tumor cells, blocks their cell cycle progression, and induces cell apoptosis [55,56]. It also indirectly activates immune cells, promotes their effector functions, or blocks their suppression in order to kill tumor cells. Previous studies have shown that IFNα increases the production and survival of CD8^+^ effector T cells [57], promotes NK cell activation and effector factor release [58], promotes B cell maturation and immunoglobulin secretion [59] and increases the antigen-presenting ability of DCs [60]. In this study, our data indicated that IFNα4 may act on other non-T cells to inhibit the growth of CRPC. We found that the proportion of G-MDSCs increased during the development of CRPC and decreased significantly following IFNα4 treatment. Furthermore, IFNα reduced the proliferation of G-MDSCs both in vivo and in vitro. It also decreased the G-MDSC-mediated inhibition of T cells. It is reported that MDSC-derived IL-23 contributed to the development of castration-resistant prostate cancer [23]. It will be interesting to investigate whether IFNα could affect IL-23 production from MDSC. Immune checkpoint antibodies have shown weak to moderate efficacy in prostate cancer [61]. It is worth testing whether IFNα could be combined with immune checkpoint antibodies to enhance the antitumor efficacy.

Although our findings are promising, our study has certain limitations. First, the TME contains many types of immune cells, and IFNα, which has a wide range of effects, may affect other immune cells as well, which we did not look at. In addition to G-MDSCs, it will be interesting to elucidate the role of IFNα on other immune cells in the prostate cancer TME, such as NK cells, macrophages, and B cells. Second, the systemic delivery of IFNα has several side effects in clinical settings. Therefore, it is critical to investigate if the targeted delivery of IFNα against a specific prostate cancer antigen or an IFNα pro-drug is more effective in reducing the side effects on non-tumor tissues.

In summary, G-MDSCs are correlated with the development of CRPC. IFNα effectively inhibits the growth of CRPC, reduces the number of G-MDSCs in tumor-bearing mice, and decreases the inhibitory effect of G-MDSCs on T cells in vitro. Our work revealed that G-MDSCs may be a potential therapeutic target, thereby presenting a new strategy for the treatment of CRPC.

## 5. Conclusions

G-MDSCs infiltration is crucial for designing immunotherapies against CRPC. IFNα promotes antitumor T cell response against CRPC by regulating G-MDSCs, thereby presenting a potential approach for the treatment of CRPC in clinical settings.

## Data Availability

Data sharing is not applicable to this article.

## Supplementary Materials

The following are available online at https://www.mdpi.com/article/10.3390/cancers13215574/s1, Table S1: Primers for RT-qPCR.
