# Supplementary material for: Type I Interferon Promotes Antitumor T Cell Response in CRPC by Regulating MDSC
Source: Cancers (Basel). 2021 Nov 8;13(21):5574. doi: 10.3390/cancers13215574 (PMC8582786; doi:10.3390/cancers13215574)
Supplement: Supplementary file 1 [file cancers-13-05574-s001.zip › cancers-1362687-supplementary.pdf]

cancers MDPI

Supplementary Materials: Type I Interferon Promotes Anti-tumor T Cell Response in CRPC by Regulating MDSC

Lilv Fan, Guiliang Xu, Jingjing Cao, Min Li, Huihui Zhang, Fanlin Li, Xinyue Qi, Xiaoqing Zhang, Zeyu Li, Ping Han and Xuanming Yang

Table S1. Primers for RT-qPCR.

Genes Names	Primer Sequence
mouse GAPDH F	AGGTCGGTGTGAACGGATTTG
mouse GAPDH R	TGTAGACCATGTAGTTGAGGTCA
mouse CD80 F	ACCCCCAACATAACTGAGTCT
mouse CD80 R	TTCCAACCAAGAGAAGCGAGG
mouse CD86 F	GGTGGCCTTTTTGACACTCTC
mouse CD86 R	TGAGGTAGAGGTAGGAGGATCTT
mouse CD40L F	TGTCATCTGTGAAAAGGTGGTC
mouse CD40L R	ACTGGAGCAGCGGTGTTATG
mouse ICOSL F	TAAAGTGTCCCTGTTTTGTGTCC
mouse ICOSL R	ATTGCACCGACTTCAGTCTCT
mouse IL-7 F	TTCCTCCACTGATCCTTGTTCT
mouse IL-7 R	AGCAGCTTCCTTTGTATCATCAC
mouse IL-15 F	ACATCCATCTCGTGCTACTTGT
mouse IL-15 R	GCCTCTGTTTTAGGGAGACCT
mouse LIGHT F	GTTTCTCCTGAGACTGCATCAA
mouse LIGHT R	TGGCTCCTGTAAGATGTGCTG
mouse 4-1BBL F	CGGCGCTCCTCAGAGATAC
mouse 4-1BBL R	ATCCCGAACATTAACCGCAGG
